# The safety and efficacy of the Stryker OptaBlate™ Bone Tumor Ablation system for vertebral body metastases

**DOI:** 10.3389/fonc.2024.1412430

**Published:** 2024-05-10

**Authors:** Alexander R. Evans, Danielle M. Harris, Mary K. Gumerlock, Hakeem J. Shakir

**Affiliations:** Department of Neurosurgery, University of Oklahoma, Oklahoma City, OK, United States

**Keywords:** Stryker, OptaBlate, spinal metastasis, radiofrequency ablation, kyphoplasty

## Abstract

**Background:**

Metastatic spinal lesions are a significant cause of morbidity and decreased quality of life in those with a high tumor burden. Despite treatment modalities such as medical therapy (e.g., chemotherapy, steroids), spinal augmentation procedures, and radiation therapy, many patients still experience refractory back pain due to neoplastic infiltration of the vertebral body and/or pathologic compression fractures. With the aim to address refractory pain in patients who have exhausted conventional treatment options, Stryker developed the OptablateTM Bone Tumor Ablation system (BTA; Stryker Corporation, Kalamazoo, MI), which delivers radiofrequency energy to pathologic vertebral body lesions. In this preliminary single-institution study, we characterize the use of the BTA system in 11 patients undergoing kyphoplasty for pathologic spinal lesions with the goal to demonstrate the impact of this novel technology on refractory pain in this challenging clinical setting.

**Methods:**

A single-center retrospective chart review was performed on all patients identified as those receiving tumor ablation/kyphoplasty for spinal neoplasms using the OptablateTM BTA system performed by a single surgeon at the University of Oklahoma Medical Center. Sex, age, primary lesion type, presenting symptomatology, spinal level, time of follow-up, and outcome were obtained from the electronic medical record (EMR).

**Results:**

Eleven patients (4 males, 7 females) with a mean age of 62 (range, 38–82) years had an average follow-up time of 6 months. Presenting symptoms attributed to spinal pathology included back pain (n = 11, 100%), pathologic fracture (n = 6, 55%), and lower extremity weakness (n = 3, 27%). A total of 20 lesions were ablated at 12 vertebral levels. Eight patients (73%) had improved pain. No complications were reported.

**Conclusion:**

This preliminary study documents the safety of the BTA system, in addition to its diverse use across many levels. The majority of patients reported improvement in their pain. Further study is required to fully characterize the use of the BTA system in those with neoplastic spinal pathology.

## Introduction

The metastasis of primary neoplasms to the spinal column is a widely prevalent phenomenon that markedly increases the risk for morbidity in patients already experiencing baseline pain secondary to a high tumor burden (1). Although metastases to the vertebral body may be asymptomatic, these lesions often present with focal back pain and/or pathologic vertebral body fractures. Current management of extradural vertebral body tumors includes medication, spinal augmentation procedures (vertebroplasty, kyphoplasty), and radiation therapy in efforts to control disease, preserve neurologic function, stabilize compression fractures, and reduce pain (2–4). Although spinal augmentation significantly decreases pain levels with minimal complications compared to open surgery (2), pain continues to be one of the most debilitating symptoms of vertebral body metastases.

To address the treatment needs of this patient population, Stryker’s OptaBlate™ Bone Tumor Ablation system (BTA; Stryker Corporation, Kalamazoo, MI) emerged with the Food and Drug Administration’s approval in 2022 (5). By ablating painful metastatic lesions to the spinal column, the BTA system now plays a role in vertebral augmentation procedures to further optimize pain control and restore vertebral body height. Further, the development of this technology offers a unique advantage of coupling bone lesion ablation with conventional augmentation procedures. In this preliminary single-institution study, we characterize the use of the Bone Tumor Ablation system in 11 patients undergoing kyphoplasty for pathologic vertebral body lesions.

## Materials and methods

A single-center retrospective chart review was performed on all patients identified as receiving tumor ablation for vertebral body neoplasms using the Optablate™ BTA system between January 12, 2023 and October 26, 2023. All procedures were performed by a single surgeon at the University of Oklahoma Medical Center Adult Patient Tower, with follow-up through March 13, 2024.

### The Bone Tumor Ablation system

**Figure 1:**
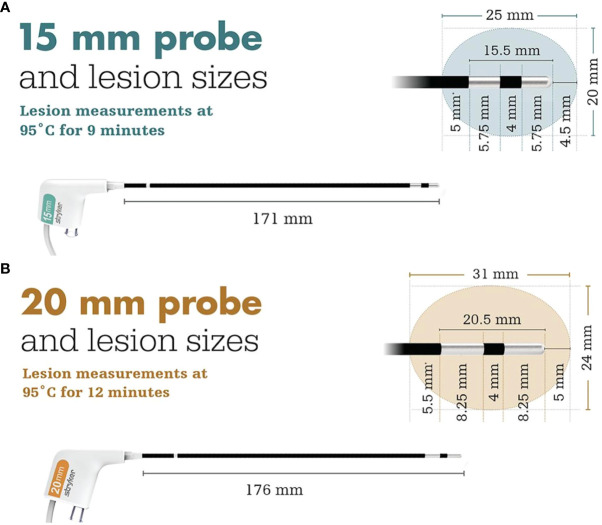
Ablation regimens and treatment area of the **(A)** 15 mm and **(B)** 20 mm probes reprinted with permission from Stryker (5).

The BTA system provides two probes for the delivery of radiofrequency ablation, depending on tumor size (**Figure 1**). Under fluoroscopic guidance, access cannulas are inserted and advanced to the area of pathology. Biopsies may then be taken for pathologic analysis. Subsequently, the cannulas are advanced to the posterior one-third of the vertebral body. A hand drill is inserted into the anterior third of the vertebral body to create a pathway through which bilateral probes are then inserted and connected to a microinfuser, allowing for the flow of sterile saline to the lesion site, thus preventing excess charring. The ablation is conducted, with surrounding tissue receiving radiofrequency energy according to prescribed regimens. Patients with lesions measuring 15mm in width receive 95°C ablation for 9 minutes, whereas patients with lesions measuring 20mm in width receive ablation at 95°C for 12 minutes (**Figure 1**) (5).

### Patient selection

Patients were selected for treatment having presented with back pain and at least one of the following: a diagnosis of focal neoplastic vertebral body lesion(s) or fracture (presumed pathologic). Exclusionary criteria included any contraindication to kyphoplasty, bleeding disorders, allergy to bone cement, or tumor mass involving the spinal canal. Patients with asymptomatic vertebral body fractures were excluded.

### Data collection and analysis

Data collection obtained from the electronic medical record (EMR) included sex, age, primary lesion type, presenting symptomatology, spinal levels treated, outcome, and time to last follow-up. Pain was assessed via direct patient questioning at follow-up, in which improvement in pain was defined as partial or complete resolution of pain symptoms. Time to follow-up was determined from procedure date to time of most recent documented hospital or clinic visit as of March 13, 2024.

## Results

### Illustrative case example

A 38-year-old woman (**Table 1**, case 6) initially presenting to the emergency department (ED) with dyspnea was found to have a distal colon mass. Upon extensive workup, she was diagnosed with primary adenocarcinoma and widespread metastases. Decision was made to pursue palliative chemotherapy for the widespread metastases and zoledronic acid therapy for bony lesions. Upon the development of midline lumbar and left hip pain, she received radiation therapy, which did not adequately control her pain despite maximum medical therapy.

**Table 1:** Summary of consecutive procedures utilizing the OptaBlate™ Bone Tumor Ablation System at our institution.

Case Number	Sex	Age	Primary Lesion Type	Presenting Symptom(s)	Spinal Level of Procedure	Outcome	Time to Follow-Up (Months)
1	F	52	SCC of the larynx	Midline lumbar pain, pathologic fracture	*L4	†Biopsy-confirmed metastatic SCC; alive at follow-up	14
2	M	52	Prostate adenocarcinoma	Lumbar pain, bowel and bladder incontinence, bilateral lower extremity weakness	L2	Died 6 months postoperatively; reported significant improvement in pain following treatment	5
3	F	72	Invasive ductal carcinoma of the breast	Lumbar pain, pathologic fracture, bilateral lower extremity weakness	T12 and L3	Died 3 months postoperatively; reported improvement in strength and pain	1
4	M	58	Renal cell carcinoma	Back/leg pain, left lower extremity weakness	L3	Alive at follow-up; reported significant improvement in pain	10
5	M	61	Multiple myeloma	Back pain with multiple pathologic fractures and lytic lesions at T7 and T9	*T7 and T9	Biopsy-confirmed plasma cell neoplasm, kappa-restricted; alive at follow-up; back pain resolved	8
6	F	38	Colorectal adenocarcinoma	Midline lumbar pain with T9/T12 lesions on imaging	T9 and T12	Died 33 days following procedure; reported immediate decrease in pain postoperatively	1 week
7	F	76	Retroperitoneal leiomyosarcoma	Midline back pain, pathologic fracture	T4	Alive at follow-up; reported resolution of pain	5
8	M	50	Colorectal adenocarcinoma	Back pain, pathologic fracture of T2, T6, T9, and T10	T9–11, L1	†Died 15 days following procedure	1 week
9	F	82	Lung, unspecified	Midline lower back pain with numerous enhancing spine lesions	T12 and L1	Died 32 days following procedure; reported a decrease in pain immediately postoperatively	2 days
10	F	64	Pancreatic adenocarcinoma	Lumbar pain	T11	Alive at follow-up; reported resolution of pain	5
11	F	78	Lung adenocarcinoma	Pathologic fracture, chronic intractable back pain	T6-T8	†Alive at follow-up	4

*Also received intraoperative biopsy.

†Pain not addressed or recorded in the patient chart.

M, male; F, female; SCC, squamous cell carcinoma.

**Figure 2:**
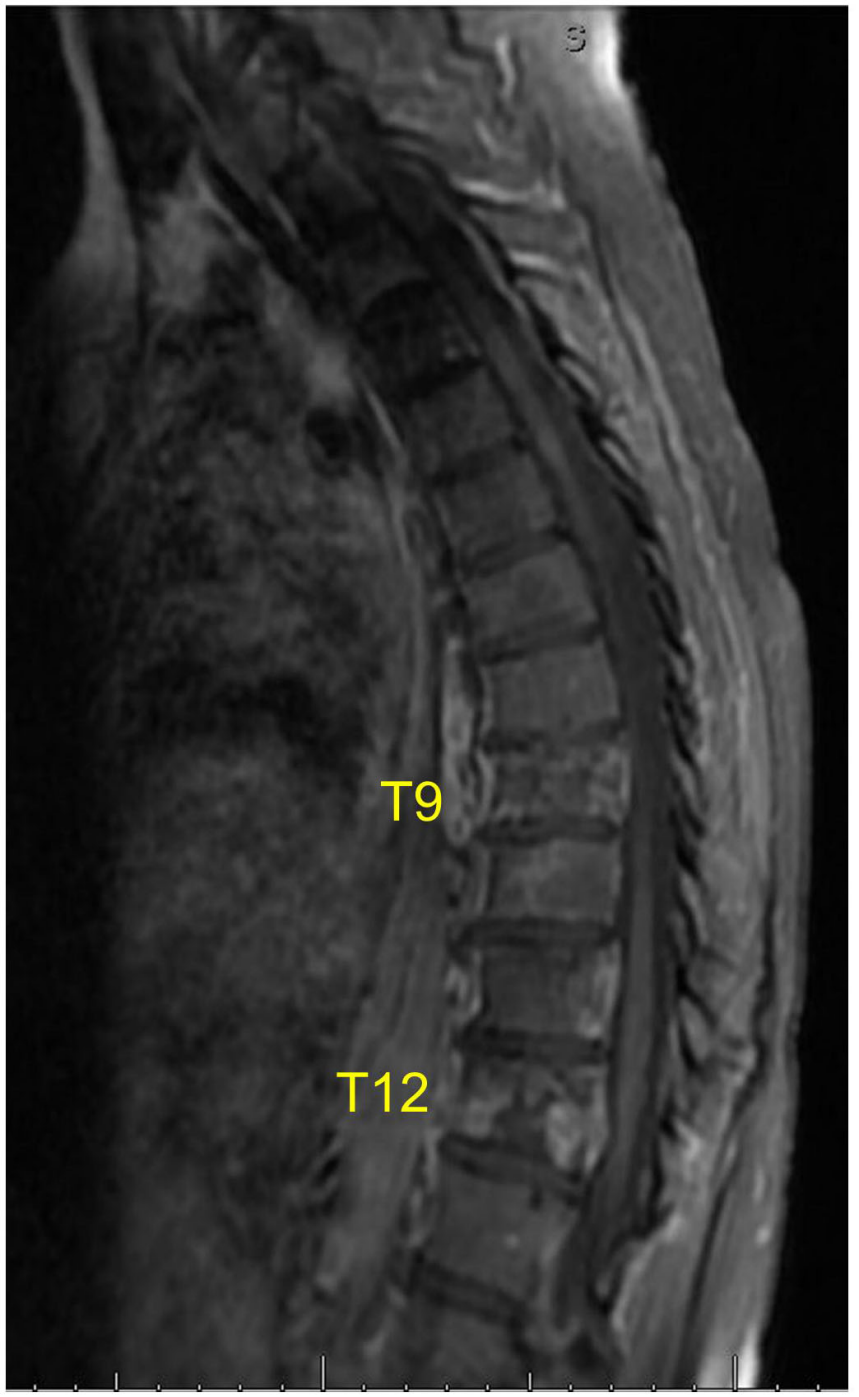
Preoperative sagittal MRI (T2-weighted) shows lesions at the T9 and T12 vertebral bodies.

Approximately one year following the patient’s initial diagnosis, she presented to the neurosurgery clinic for the evaluation of refractory back pain. At that time, MRI of the spine revealed new metastatic infiltration and compression of the T9 and T12 vertebral bodies (**Figure 2**). Given her extensive history of pain, the decision was made to perform radiofrequency ablation and kyphoplasty of these neoplastic lesions with the goal of palliation.

**Figure 3:**
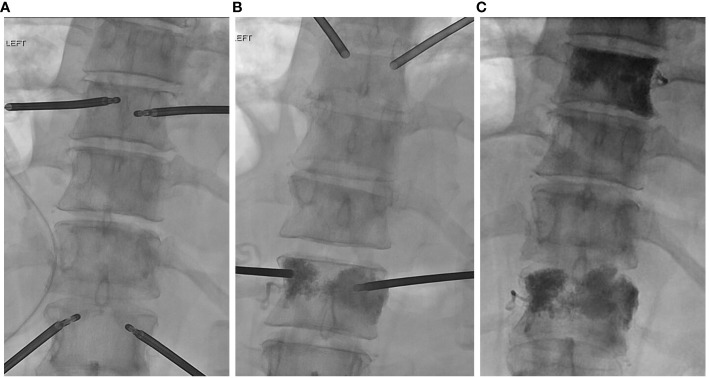
**(A)** Insertion of transpedicular ablation probes prior to bone tumor ablation and **(B, C)** bone cement placement following ablation at the T9 and T12 spinal levels.

The procedure was conducted under general anesthesia. Following localization at the proper spinal levels, stab incisions were made over the lateral pedicles. Trocars (11 gauge) provided pedicular access to the vertebral bodies. A hand drill was then used to enter the vertebral body near the anterior cortex. The OptaBlate™ 15mm radiofrequency ablation probe, inserted through the bilateral T9 and T12 pedicles to the lesions in the vertebral bodies (**Figure 3A**), delivered 95°C for 9 minutes at each site. Following ablation, kyphoplasty balloons were placed at each site and inflated to create a cavity. Bone cement was then placed into each vertebral body in 0.5ml aliquots (**Figures 3B, C**), thus concluding the kyphoplasty portion of the procedure. No postoperative complications were observed. The patient recovered well and was discharged home on the same day. She reported a noticeable improvement of pain symptoms at 7 days post-procedure and was comfortable until her death 33 days later.

### Study results

Eleven patients (4 males, 7 females) with a mean age of 62 (range, 38–82) years had an average follow up of 6 months. All patients had vertebral body metastases from a primary neoplastic process, including retroperitoneal leiomyosarcoma, pancreatic adenocarcinoma, prostate adenocarcinoma, invasive ductal carcinoma of the breast, renal cell carcinoma, lung adenocarcinoma, squamous cell carcinoma (SCC) of the oropharynx, lung carcinoma, multiple myeloma, and colorectal adenocarcinoma (**Table 1**). Presenting symptoms attributed to vertebral pathology included back pain (n = 11, 100%), pathologic fracture (n = 6, 55%), and lower extremity weakness (n = 3, 27%).

**Figure 4:**
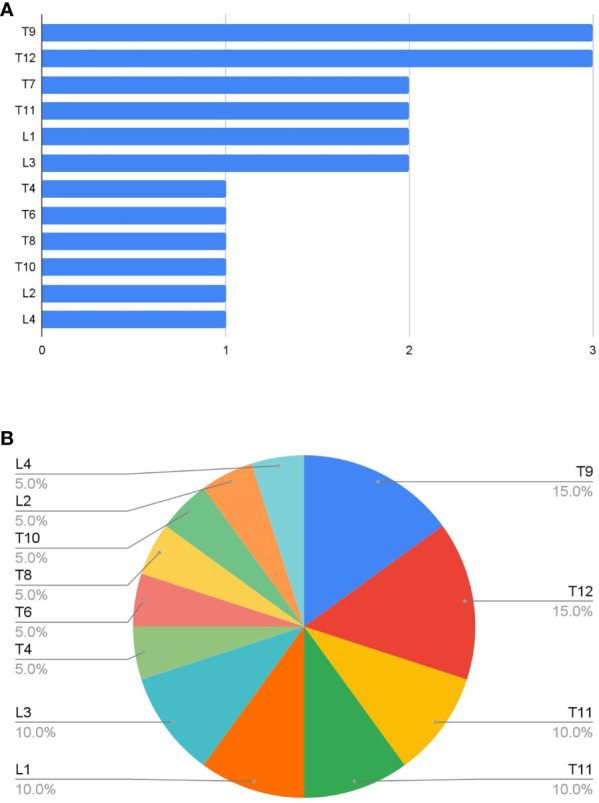
**(A)** Number and **(B)** proportion of lesions ablated at each vertebral level. T9, T12, L1, and L3 spinal levels were the most frequent sites treated.

A total of 20 lesions were ablated in 11 patients (**Figure 4**). Two patients had concurrent biopsy and all patients received ablation followed by kyphoplasty with the use of Stryker kyphoplasty balloons and Stryker bone cement. Eight patients (73%) had improved pain as documented on chart review. No complications were reported (**Table 1**).

## Discussion

In this pilot study, a total of 20 vertebral body lesions in 11 patients were treated across 12 spinal levels, ranging from T4 to L4. The majority of patients (73%) had improvement of pain following ablation and kyphoplasty, as documented at follow-up. In addition, the Optablate Bone Tumor Ablation System provided technical success in 100% of patients, with no complications observed. Within the context of extensive malignant pathology, the safety and diverse application of the BTA system in conjunction with spinal augmentation procedures significantly improved pain management in those with disease.

Historically, palliative radiation has demonstrated significant pain control in patients with painful vertebral body lesions (4). In addition, corticosteroids have been established as an effective method of decreasing pain while preserving neurological function (3). In cases of refractory pain, tricyclic antidepressant and anticonvulsant medications are often utilized, with opioids now used more frequently for breakthrough pain (3). In light of the ongoing opioid epidemic in the United States and continued lack of pain control in this patient population, novel treatment options are needed for this therapeutically challenging clinical scenario.

A multi-modal approach to vertebral body metastases is emphasized in the present study, as the majority of patients had decreased pain following the combination of ablation with vertebral augmentation. Alternatively, cases of spinal instability and cord compression more frequently necessitate surgical intervention (6). If pain is related to instability, ablation with kyphoplasty may be advantageous by eliminating underlying pathology while restoring the structural integrity of the vertebral body.

In clinical circumstances where pain control is the predominant goal, the combination of steroids and radiotherapy is often the initial therapeutic strategy, with surgical augmentation reserved for cases of metastasis-related vertebral body fractures (4, 7). Interestingly, novel stereotactic radiosurgery has emerged as an initial treatment in those with metastatic cord compression, and has shown lower rates of failure than the combination of augmentative surgery followed by radiosurgery (8). Despite the evolving therapeutic landscape of surgical and/or medical management of vertebral metastases, refractory back pain remains as one of the most prevalent and debilitating symptoms of lesions in the spinal column.

Stryker’s BTA system offers an innovative method of decreasing pain in this patient population. Whereas the boundaries of surgical augmentation, radiation therapy, and medical management have been explored, the novel BTA system couples lesion ablation with spinal augmentation. With the absence of complications in this pilot study, the BTA system appears to provide excellent pain control while maintaining a high level of safety when used in concordance with kyphoplasty. Although prior methods of analgesia have proved beneficial, the BTA system may be employed as an alternative or adjunct option for cases of particularly refractory or debilitating spinal pain. In addition, maintaining vertebral body height with kyphoplasty may place patients at a lower risk for the subsequent development of neurological deficits. Potential complications must be acknowledged, encompassing those observed in general radiofrequency ablation and kyphoplasty procedures. These include postablation radicular pain (secondary to heat-induced nerve damage), nerve root injury, cement embolism or extravasation, and infection (9–11). Selection of the proper ablation probe and adherence to the predetermined time and temperature regimen based on tumor size is critical in preventing ablation-related damage to the surrounding anatomy. We select a transpedicular route rather than a posterolateral one, to avoid nerve root injury (10). Regarding the prevention of cement embolism or extravasation, the use of high quality fluoroscopy may be advantageous by allowing clear visualization of cement progression and avoidance of venous structures in real time (10, 11). In the context of infection, polymerase chain reaction (PCR) may be used to promptly identify the infectious organism for tailored antibiotic management, in which surgical debridement may also be indicated (11).

This preliminary retrospective study has multiple limitations, including small sample size (n=11) and follow-up. Due to the progressive nature of metastatic disease, shortened follow-up time was a significant source of bias, as three patients died shortly following the procedure. Bias was also introduced from the subjective reporting of decreased pain in each patient’s record. Ideally, numerical scoring methods would have been collected in order to quantify pain relief; however, the retrospective nature of this study revealed inconsistencies in the reporting of pain and quality of life scores. For example, seven patients had Numeric Rating Scale measurements, two had Karnofsky Performance Status, and seven had Eastern Cooperative Oncology Group (ECOG) scores reported pre- and postoperatively in the medical record. In addition, our conclusions are limited to patients undergoing concurrent kyphoplasty, in which the prior treatment of metastatic neoplasms were widely variable between patients (three patients received spinal radiation, and many others received a diverse range of chemotherapeutic agents). Considering that kyphoplasty significantly improves pain in those with painful vertebral body metastases, future works may consider comparing pain outcomes in those receiving ablation with kyphoplasty versus those undergoing kyphoplasty alone.

Quality of life in patients with painful pathologic lesions and/or vertebral body compression fractures is significantly decreased. Current adjunct treatment of vertebral metastases includes radiation and spinal augmentation procedures, often without complete resolution of symptoms. In this preliminary study, we have documented the safety of the BTA system in treating pain across frequently-involved vertebral levels often the site of metastatic disease. Overall, the combination of lesion ablation and kyphoplasty is safe and can result in significant quality of life improvement in patients with pathologic vertebral body lesions. Future directions should include standardized prospective studies to more definitively establish the impact of the BTA system on pain in those with vertebral lesions.

## Data availability statement

The raw data supporting the conclusions of this article will be made available by the authors, without undue reservation.

## Ethics statement

The studies involving humans were approved by The University of Oklahoma Health Sciences Center Institutional Review Board. The studies were conducted in accordance with the local legislation and institutional requirements. The participants provided their written informed consent to participate in this study.

## Author contributions

AE: Data curation, Writing – original draft, Writing – review & editing. DH: Writing – original draft. MG: Writing – review & editing. HS: Conceptualization, Investigation, Writing – review & editing.
